# Gibberellin Deficiency Confers Both Lodging and Drought Tolerance in Small Cereals

**DOI:** 10.3389/fpls.2016.00643

**Published:** 2016-05-13

**Authors:** Sonia Plaza-Wüthrich, Regula Blösch, Abiel Rindisbacher, Gina Cannarozzi, Zerihun Tadele

**Affiliations:** ^1^Institute of Plant Sciences, University of BernBern, Switzerland; ^2^Institute of Biotechnology, Addis Ababa UniversityAddis Ababa, Ethiopia

**Keywords:** drought tolerance, *Eragrostis tef*, finger millet, GA inhibitor, lodging tolerance, paclobutrazol (PBZ), rice

## Abstract

Tef [*Eragrostis tef* (Zucc.) Trotter] and finger millet [*Eleusine coracana* Gaertn] are staple cereal crops in Africa and Asia with several desirable agronomic and nutritional properties. Tef is becoming a life-style crop as it is gluten-free while finger millet has a low glycemic index which makes it an ideal food for diabetic patients. However, both tef and finger millet have extremely low grain yields mainly due to moisture scarcity and susceptibility of the plants to lodging. In this study, the effects of gibberellic acid (GA) inhibitors particularly paclobutrazol (PBZ) on diverse physiological and yield-related parameters were investigated and compared to GA mutants in rice (*Oryza sativa* L.). The application of PBZ to tef and finger millet significantly reduced the plant height and increased lodging tolerance. Remarkably, PBZ also enhanced the tolerance of both tef and finger millet to moisture deficit. Under moisture scarcity, tef plants treated with PBZ did not exhibit drought-related symptoms and their stomatal conductance was unaltered, leading to higher shoot biomass and grain yield. Semi-dwarf rice mutants altered in GA biosynthesis, were also shown to have improved tolerance to dehydration. The combination of traits (drought tolerance, lodging tolerance and increased yield) that we found in plants with altered GA pathway is of importance to breeders who would otherwise rely on extensive crossing to introgress each trait individually. The key role played by PBZ in the tolerance to both lodging and drought calls for further studies using mutants in the GA biosynthesis pathway in order to obtain candidate lines which can be incorporated into crop-breeding programs to create lodging tolerant and climate-smart crops.

## Introduction

Tef [*Eragrostis tef* (Zucc.) Trotter] and finger millet [*Eleusine coracana* Gaertn] are small-grain cereal crops largely cultivated in developing countries. While, tef is a staple crop mainly in Ethiopia, where it is annually cultivated on about three million hectares of land with a total production of 4.7 million tons (Central Statistics Agency [CSA], 2014), finger millet is cultivated in 25 countries in Africa and Asia producing about 4.5 million tons (NAP, 1996). Both tef and finger millet are known to be tolerant to extreme climatic and soil conditions; hence, they are favorite crops in semi-arid areas with moisture limitations (Tadele and Assefa, 2012). The absence of gluten in the tef grain (Spaenij-Dekking et al., 2005) makes it a life-style crop at the global level (Provost and Jobson, 2014). Due to these health-related benefits, the cultivation and consumption of tef has increased tremendously in recent years outside Ethiopia. The seeds of finger millet contain valuable amino acids especially methionine (NAP, 1996), which is lacking in the diets of hundreds of millions of the poor who live on starchy staples such as cassava. Finger millet is also a popular food among diabetic patients because of its low glycemic index and slow digestion (Chandrashekar, 2010). The straw from both tef and finger millet is a valuable source of livestock feed especially the one from tef which is more palatable and nutritious than that from wheat and barley (Yami, 2013).

Despite these desirable traits, both tef and finger millet have inferior yields. In Ethiopia, the average yield in the 2013 cropping season was only 1.4 ton ha^-1^ for tef and 1.8 ton ha^-1^ for finger millet as compared to wheat (2.1 ton ha^-1^) and maize (3.1 ton ha^-1^; Central Statistics Agency [CSA], 2014). The low productivity of these crops was mainly due to the unavailability of improved cultivars. Although both tef and finger millet are moderately tolerant to moisture scarcity compared to wheat and maize, considerable amounts of grain yield and biomass of tef and finger millet are lost annually due to drought as both crops are mostly allocated to semi-arid areas with substantial moisture deficiency during the growing season (Degu et al., 2009; Assefa et al., 2011; Tadele, 2016). The effect of drought on tef productivity depends on the developmental stage at which the stress occurs. A yield loss of up to 53% was reported when the drought occurred at the grain filling period indicating that the most susceptible stage for tef is terminal drought (Mengistu, 2009).

Lodging, the permanent displacement of the plant from the vertical position, is also a major bottleneck in tef production due to the tall and slender nature of the plant. In tef, lodging frequently occurs just before the ripening of the grain, resulting not only in inferior grain yield but also in shriveled and poor quality seed that sprouts on the panicle before harvest. Among the two commonly known lodging types in plants, namely stem- and root-lodging, tef was shown to be more susceptible to root lodging (van Delden et al., 2010). Lodging is also among the major yield limiting factors in finger millet production (Degu et al., 2009).

Plant growth regulators (PGRs) have been used in crop production to reduce plant height and, as a result, to improve lodging tolerance. Gibberellic acid (GA) inhibitors play key role in developing semi-dwarf and sturdier plants against lodging (Rademacher, 2000; Berry et al., 2004). The reduction in plant height is associated with shorter internodes (Sanvicente et al., 1999). The alteration or deficiency of GA was key in developing semi-dwarf and high yielding wheat and rice cultivars during the famous Green Revolution which boosted both the production and productivity of the two cereals (Hedden, 2003).

Gibberellic acid inhibitors which have been widely utilized to regulate the architecture of plants include paclobutrazol (PBZ), chlormequat-Cl, mepiquat-Cl, and daminozide. PBZ [α-*tert*-Butyl-β-(4-chlorobenzyl)-1*H*-1,2,4-triazole-1-ethanol] is a triazole which inhibits the conversion of the GA precursor *ent*-kaurene to *ent*-kaurenoic acid (Hedden and Graebe, 1985). PBZ is known to cause several physiological changes including the reduction of plant height, improved nutrient uptake and enhanced seed yield (Davis et al., 1988). The application of PBZ to Japanese paddy rice was responsible for reducing the plant height by 90% and lodging by 60% while raising the yield by 15% (French et al., 1990). Chlormequat-Cl [2-chloroethyltrimethylammonium chloride], also known as CC, and mepiquat-Cl [piperidinium, 1,1-dimethyl-, chloride], or MC, have been used as plant regulators for several decades. These two inhibitors block the GA biosynthesis by inhibiting the cyclization of geranylgeranyl diphosphate (GGPP) to *ent*-copalyl diphosphate (CPP; Rademacher, 2000). While Chlormequat-Cl is known to delay the lodging period in wheat, barley, and oat (Clark and Fedak, 1977), mepiquat-Cl shortened the internodes in cotton and therefore resulted in semi-dwarf plants (Mccarty and Hedin, 1994). On the other hand, daminozide [succinic acid 2,2-dimethyl hydrazide] (DM), a co-substrate of dioxygenases that catalyzes the last step of GA formation, reduced excessive vegetative growth in trees and ornamental plants (Rademacher, 2000).

Although GA biosynthesis inhibitors, specifically PBZ, are known to reduce plant height and increase lodging tolerance in cereals such as rice and wheat, no link between lodging tolerance and drought tolerance especially in relation to GA inhibitor(s) has been reported.

The aim of the current investigation was, therefore, to determine the effect of GA inhibitors on important agronomic traits in tef and finger millet, specifically to lodging- and drought- tolerance. In addition, semi-dwarf rice mutants deficient or defective in the GA biosynthesis pathway (Okuno et al., 2014) were evaluated to determine if they also respond differently to moisture scarcity.

## Materials and Methods

### Plant Materials and Growing Conditions

Two improved tef varieties, namely *Dukem* (DZ-01-974) and *Tsedey* (DZ-Cr-37) were used in diverse experiments. Seeds of tef germinated in the long-day room (16 h light at 22°C and 65% RH and 8 h dark at 18°C and same RH) and were transferred after 3 weeks to the short-day room (8 h light at 22°C and 65% RH, and 16 h dark at 18°C and same RH). Seeds of finger millet were germinated in the long-day room for 2 months before being transferred after the water deficit (WD) treatment to an equal-day room (12 h light at 25°C and 65% RH, and 12 h dark at 19°C and 65% RH). Seeds of rice lines *Ginbozu*, *T65* and their respective mutants, namely *Tan-Ginbozu* and *gid1-8* (Okuno et al., 2014; gifts from Dr. Ko Hirano, Nagoya University, Japan) were maintained for all experiments in the equal-day room.

The soil consisted of five parts of topsoil, four parts of turf, and two parts of quartz sand and fertilized with a Hauert Plantaktiv 16+6+26 type K fertilizer (Hauert HBG Dünger Schweiz, Grossaffoltern, CH) which contains 16% nitrogen, 6% phosphate, 26% potassium, 2% magnesium, and micronutrients (0.02% borate, 0.04% copper, 0.1% iron, 0.05% manganese, 0.01% molybdenum, and 0.01% zinc). The photosynthetic photon flux (PPF) measured by a quantum meter (Apogee Instruments, Logan, UT) was 127.5 ± 4.5 and 168.5 ± 4.5 μmol m^-2^ s^-1^ under short- and long-day conditions, respectively.

### GA Inhibitors and Abiotic Stresses

Seeds were surface sterilized for 10 min in commercial bleach (2.5% NaClO), followed by several rinses with sterile water. The seeds were sown on agar plates with ½ MS medium (Murashige and Skoog, 1962) and placed at 21°C in the dark for 8 h and 25°C in the light for 16 h with a continuous humidity of 50%. The length of the shoots and roots were measured after 9 days. Treatments used for the four GA inhibitors were chlormequat-Cl or CC (0, 1000, 1500, and 2000 mg l^-1^), daminozide or DM (0, 100, 500, 1000, and 1500 mg l^-1^), mepiquat-Cl or MC (0, 500, 1000, 1500, and 2000 ppm), and PBZ (0, 0.1, 1, 10, and 100 μM). For the transfer or recovery assays, seedlings grown for 4 days on 100 μM PBZ plates were transferred to a medium with or without 5 μM GA.

The effects of PBZ on abiotic stresses were investigated by first growing seedlings on a medium containing 10 μM PBZ followed by transferring to the medium containing different types and levels of abiotic stresses. These include mannitol (0, 200, 400, and 600 mM), NaCl (0, 100, 150, 200, and 250 mM), ABA (0, 0.5, 1, 2, and 3 μM), and H_2_O_2_ (0, 0.2, 0.5, 1, and 1.5 mM). The effects of PBZ application to the response of tef plants to cold or heat treatment were investigated by subjecting 3-day old seedlings to either 4°C (for cold) or 40°C (for heat) for 1, 6, 24, 48, or 72 h.

For plants grown on soil, PBZ (0 and 100 μM) was sprayed a total of six times (100 μl per spray per plant) for tef and three times (100 μl per spray per plant) for finger millet at 2-day intervals starting on 10-day-old plants.

### Growth and Related Parameters

Number, length, and diameter of internodes per culm as well as number of tillers were measured for main culms of 1-month old plants. Length, width, and density of cells were determined using a scanning electronic microscope (SEM) pictures analyzed by ImageJ (WSR, 2015) for five different plants per treatment. Numbers of cells in each internode were determined as follows: cell number mm^-2^ × Pi × inter node length × (inter node radius)^ˆ2^. At harvesting time, yield-related parameters such as plant height, number of tillers and panicles, shoot biomass and grain yield were recorded. Shoot dry biomass was determined from samples dried overnight at 60°C.

### Lodging Tolerance

Lodging of tef plants was quantified using the angle of displacement of the stem from the vertical position where 0° is no displacement and 90° is completely horizontal. Each plant was photographed and the angle determined using the ImageJ software (WSR, 2015) at the heading and maturity stages.

### Tolerance to Moisture Deficit

Transpiration was determined by measuring the amount of water lost through stomata from plants grown in sealed pots. Pots were water saturated at the beginning of the experiment and no additional moisture was added during the entire experimental period for treatments with WD while plants watered every 3 days for well-watered (WW) conditions. Transpiration was calculated as the difference of weight in grams compared to the starting point minus the amount lost by the control plant. Stomatal data such as conductance, length, width, and density were measured at the end of the experiment. Moreover, shoot- and root-biomass were quantified at the end of the water stress period. Stomatal conductance of both the adaxial (the top of the leaf) and the abaxial (the bottom of the leaf) side of the flag leaf was determined using an AP4 diffusion porometer (Delta T, Cambridge Life Sciences, Cambridge, UK). Stomatal size was determined by SEM microscopy. In addition, chlorophyll a and b as well as carotenoids which comprise carotenes and xanthophylls were extracted using 95% ethanol as previously described (Lichtenthaler, 1987). The content of these pigments were normalized by dry weight.

A recovery assay was made by imposing WD on tef and finger millet for 1 week and rice for 10 days, followed by normal watering until harvesting. Dehydration was done on 6 week-old finger millet and 2 month-old rice plants. Percentage of rolling leaves was determined at the end of the water stress and after 3 and 7 days of re-watering. Moreover, water content of shoot and root defined as (FW – DW)/FW was measured at the end of the water stress; where FW, fresh weight; and DW, dry weight. For finger millet and rice, stomatal conductance was determined at the end of the water stress treatment.

### Gene Expression Analysis with Quantitative Real-Time PCR

The *Tsedey* tef variety was sown and grown until the water stress treatment was applied. One hundred milligrams of leaf tissue was used to isolate total RNA with a Promega kit followed by DNase I (Promega) treatment for 15 min at 37°C. First-strand cDNA synthesis was performed using M-MLV reverse transcriptase (Promega). Second-strand synthesis was conducted with the LightCycler^®^ 96 System (Roche) using the FastStart Essential DNA Green Master Kit (Roche). Fifty cycles of PCR were made at 95°C for 10 s, 61°C for 10 s, and 72°C for 12 s using *KO2* specific primers from the GA biosynthesis pathway. To calculate the relative gene expression, the 2^-ΔΔ^*^Cq^* method was used (Livak and Schmittgen, 2001). *C*q-values were given as the mean of three replicates. As a reference, the constitutively expressed α-Tubulin 1 gene was used. The sequences of the two sets of primers from the tef genome (Cannarozzi et al., 2014) were:

*Tua1*-Forward: 5′-TGTCTCAGGTCATCTCATCAC-3′

*Tua1*-Reverse: 5′-GTAGGAGGAAAGCATGAAGTGG-3′

*KO2*-Forward: 5′-AGTTGGTAATGCTGGTGTGG-3′

*KO2*-Reverse: 5′-AAGGCGATCTTGTTTCTCTGG-3′

### Statistical Analysis

Statistical tests were made using SPSS 17.0 (IBM, Chicago, IL, USA). Significant differences between treatment means were determined using non-parametric tests (Mann–Whitney *U* or Kruskal–Wallis *H* tests) and a *p*-value of ≤0.05. For pot experiments, two replicates of five single plants were analyzed, whereas for MS plates, two replicates with 6–7 seedlings per plate were used. For the qRT-PCR, three independent samples per treatment were analyzed.

## Results

### GA Inhibitors Reduced the Growth of Tef Plants

**FIGURE 1:**
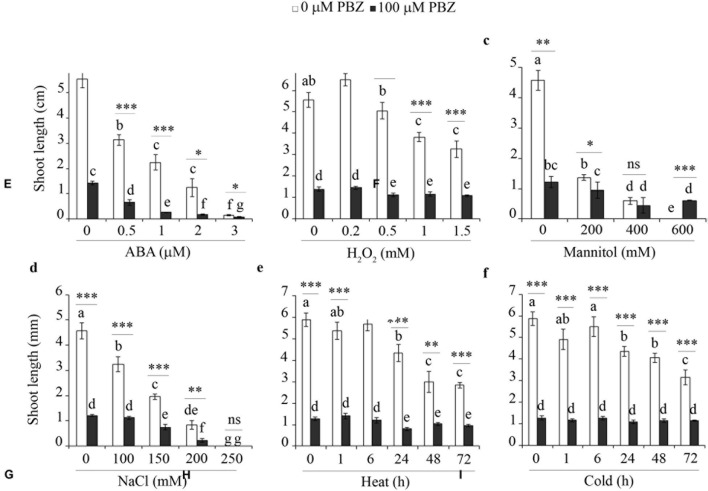
**Four gibberellic acid (GA) inhibitors substantially reduced the growth of tef seedlings**. Tef seedlings treated with different levels of chlormequat-Cl or CC **(A)**, daminozide or DM **(B)**, mepiquat-Cl, or MC **(C)**, and paclobutrazol or PBZ **(D)**. The length of shoots **(E)** and roots **(F)** of seedlings treated with different levels of the four GA inhibitors. PBZ treated plants partially recovered after transfer to medium with gibberellic acid **(G)**. Shoot length **(H)**, and root length **(I)** of plants that recovered from the PBZ effect. Arrows in **(H,I)** indicate the date of transfer to the GA medium. Means with different letters were significantly different from each other at *p* < 0.05.

The increasing levels of all tested GA inhibitors, namely CC, DM, MC, and PBZ substantially hindered the growth of tef plants at the early developmental stage (**Figures 1A–D**). These considerable negative effects on growth were observed for both the shoots and roots of tef plants treated with all four GA inhibitors (**Figures 1E,F**). Among the four GA inhibitors, strong and consistent effects on the shoots of tef seedlings were observed by PBZ. Hence, further investigations on morphological, mechanical and physiological properties of tef were performed using PBZ. In order to confirm that the drastic height reduction in tef seedlings was due to GA inhibition, tef seedlings grown for the first 4 days on a medium with PBZ were transferred to a medium with GA. As shown in **Figures 1G–I**, seedlings placed on the GA medium recovered from the drastic effect of PBZ although the recovery was not to the level observed for the control plants without GA inhibitor. Significant recovery was observed for shoots only 2 days after removal from the PBZ medium (**Figure 1H**). Recovery of roots from PBZ treatment was not significant, even 5 days after transferring to the GA medium (**Figure 1I**).

### PBZ Reduced Plant Height and Improved Lodging Tolerance

**FIGURE 2:**
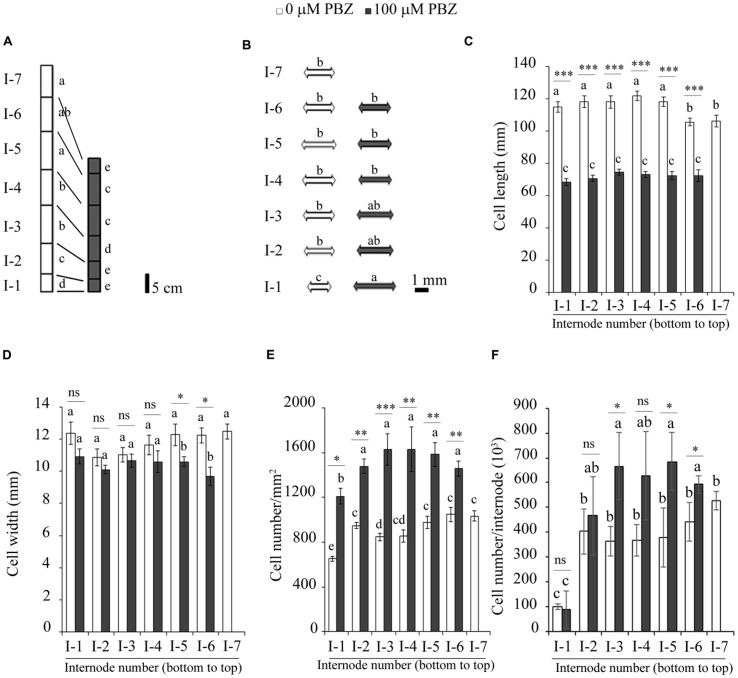
**PBZ reduced the height of tef plants by affecting the length and number of internodes and cells. (A)** The length and number of internodes of PBZ treated and untreated plants. **(B)** The average diameter of internodes of PBZ treated and untreated plants. Cell length **(C)**, cell width **(D)**, number of cells per mm^2^ **(E)** and number of cells per internode **(F)** for PBZ treated and untreated plants. Means with different letters were significantly different from each other at *p* < 0.05. ^∗∗∗^*p* < 0.001; ^∗∗^*p* < 0.01; ^∗^*p* < 0.05; ns, no significant difference.

Paclobutrazol reduced the height of tef plants by decreasing the number and length of the internodes (**Figure 2A**). On the average, the tef variety used in the present study, *Tsedey*, has seven internodes (I-1 to I-7), but PBZ application reduced the number of internodes to six. Surprisingly, the internodes at the bottom of the PBZ treated plants were significantly wider than those of the untreated ones (**Figure 2B**). Detailed measurements at the cellular level showed that PBZ substantially reduced the cell length (**Figure 2C**) but had little effect on the cell width (**Figure 2D**). These extremely small cell lengths in the PBZ treated plants were also reflected in elevated numbers of cells per unit area and per internode (**Figures 2E,F**). Patterns in the length and width of cells were mainly unchanged among different internodes for both PBZ treated and untreated plants indicating that the inhibitor had an overall homogenous effect at the whole plant level. Similar to tef, the application of PBZ significantly reduced the height of finger millet seedlings, for which up to 90% height reduction was obtained (Supplementary Figure S1).

**FIGURE 3:**
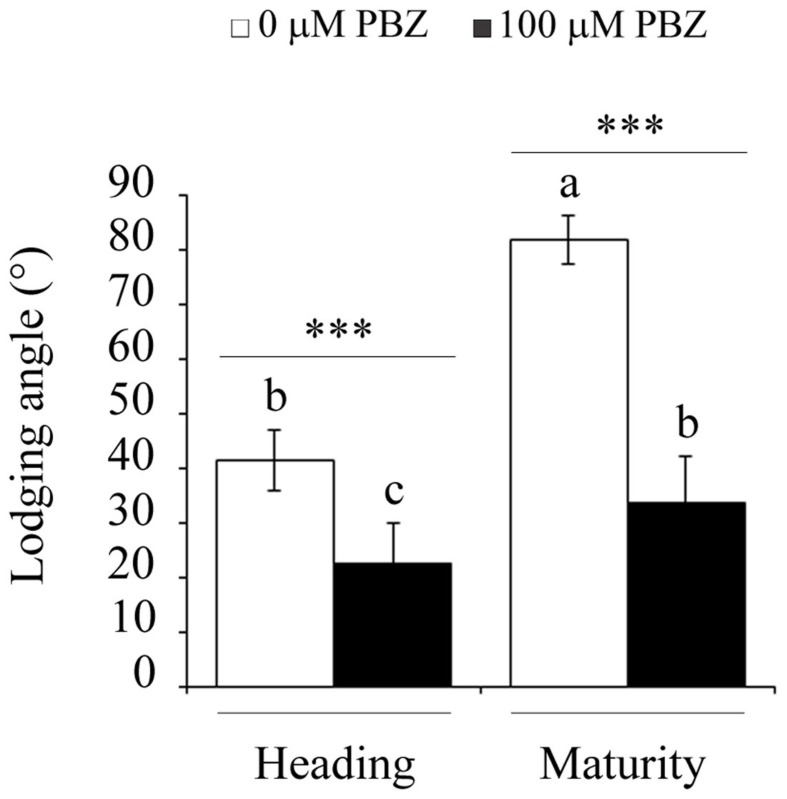
**PBZ treated tef seedlings showed strong resistance to lodging both at the heading and maturity stages**. Different letters refer to significant differences among the group of treatments while an asterisk refers to a highly significant difference (^∗∗∗^*p* < 0.001) between PBZ treated and PBZ untreated samples.

The reduction of the height of the plant by treatment with PBZ decreased lodging in tef plants (**Figure 3**). Lodging, defined as the angle of deviation of the main stem from the vertical position, was significantly lower for PBZ treated plants compared to untreated ones at both the heading and maturity stages. Compared to control plants, in PBZ treated plants, lodging decreased by 40% at the heading, and by 60% at the maturity stages. This shows that PBZ did not only reduce the height of the plant but also made the plant sturdier or tolerant against lodging.

### PBZ Enhanced Drought Tolerance in Tef and Finger Millet

**FIGURE 4:**
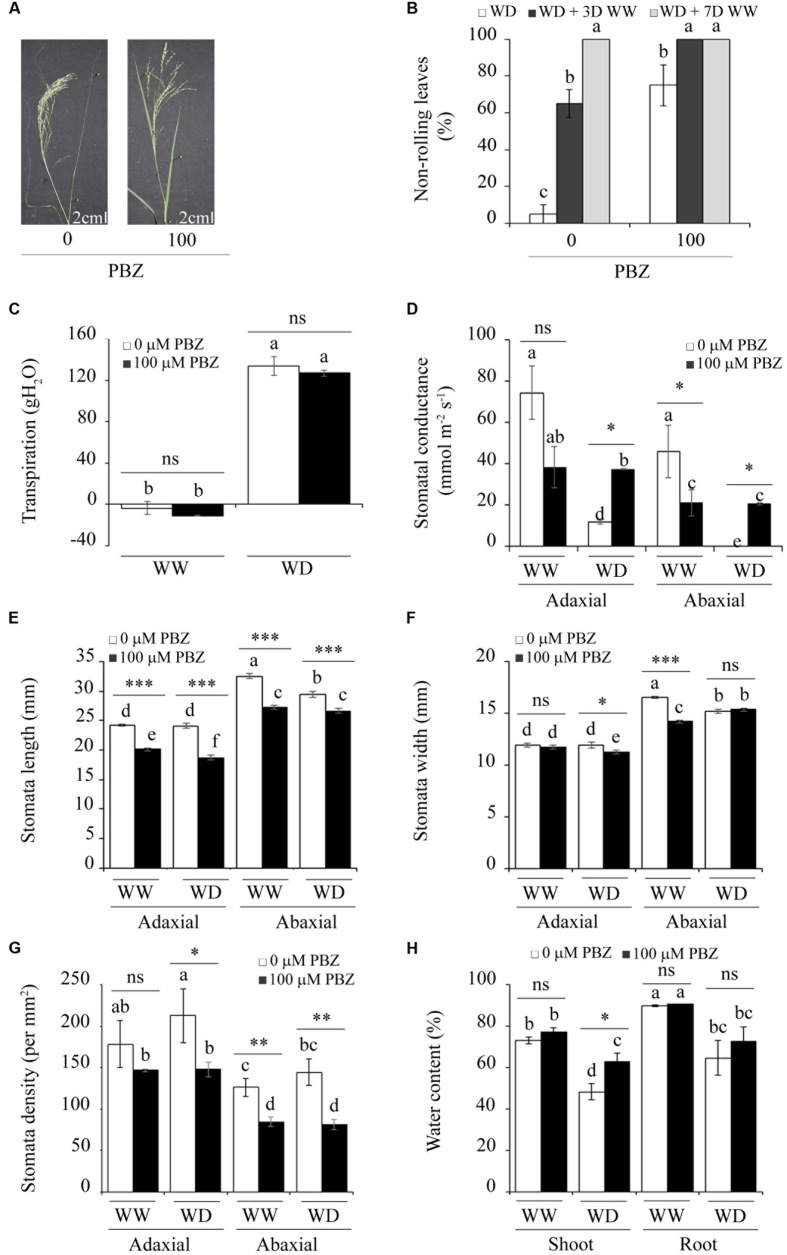
**PBZ enhanced the tolerance of tef to drought through changes in the morphology and physiology of the plant. (A)** PBZ untreated plants exposed to moisture deficiency revealed a strong leaf-rolling phenotype compared to PBZ treated ones. **(B)** The proportion of non-rolling leaves for PBZ treated and untreated plants under drought (WD, water deficit), drought followed by 3 days (WD + 3WW), and 7 days of watering (WD + 7WW). Transpiration **(C)**, stomatal conductance **(D)**, stomatal length **(E)**, stomatal width **(F)**, stomatal density **(G)**, and plant water content **(H)** for normally watered (WW, well-watered) and WD conditions. Different letters refer to significant differences among the group of treatments while an asterisk refers to a significant difference between PBZ treated and PBZ untreated samples. ^∗∗∗^*p* < 0.001, ^∗∗^*p* < 0.01, ^∗^*p* < 0.05; ns, no significant difference.

The effects of moisture scarcity could be assessed by symptoms observed on plants exposed to the stress. The proportion of leaf rolling and physiological parameters such as osmotic adjustment and stomatal aperture have been used as criteria to study drought tolerance in monocot plants (Otoole and Cruz, 1980; Balsamo et al., 2006; Lopes et al., 2011). The intensity of leaf rolling (or pin-shaped leaves) indicates the severity of drought stress on the plant (**Figures 4A,B**). PBZ treated plants and those exposed to moisture deficit had only 30% leaf rolling. However, PBZ untreated but exposed to drought had over 90% leaf rolling (**Figure 4B**). Although both PBZ treated and untreated tef seedlings recovered fully from the drought treatment, those treated with PBZ recovered only within 3 days compared to PBZ untreated plants, which took 7 days. Investigations on transpiration, stomata, and plant water content provided clues about the effects of PBZ on the morphological and physiological parameters involved in drought-related responses (**Figure 4C**). Irrespective of PBZ application, tef plant lost over 100 g water when exposed to moisture scarcity.

Under the normal moisture regime, both the adaxial and abaxial parts of PBZ untreated plants displayed higher stomatal conductance than the corresponding parts from plants treated with PBZ (**Figure 4D**). However, under the low moisture regime, plants treated with PBZ had significantly higher stomatal conductance at both sides of the leaf compared to untreated ones. Among the morphological parameters of stomata that were investigated (**Figures 4E,F**), stomatal length was the most interesting as PBZ treated plants had significantly (*p* < 0.001) shorter stomata under both moisture regimes and both sides of the leaf compared to PBZ untreated plants (**Figure 4E**). This substantial and consistent reduction in the length of stomata due to PBZ application might be the reason for the enhanced drought tolerance in PBZ treated plants although the transpiration rate was similar between the two groups of plants under drought conditions (**Figure 4C**). The effect of PBZ on stomata width was not conclusive since significant and negative effects were obtained due to PBZ application on the adaxial side of the leaf under moisture limited conditions and on the abaxial side for moisture unlimited conditions (**Figure 4F**). Furthermore, in PBZ treated plants, a substantial reduction in the stomatal density was obtained at both the adaxial and abaxial sides of the leaf and for both moisture regimes (**Figure 4G**). The stomatal density was higher on the adaxial side than the abaxial side for both PBZ treated and untreated plants. Regarding the plant water content, the only significant change was obtained from shoots exposed to drought, in which PBZ treated plants had higher moisture content in their above-ground part (**Figure 4H**).

Paclobutrazol sprayed finger millet plants were not only dwarf but also tolerant to moisture deficit as was shown with green leaves (Supplementary Figure S2a). In finger millet, PBZ clearly protected the flag leaf from rolling (Supplementary Figure S2b) and all plants had recovered after only 1 day of rehydration (*n* = 10). For PBZ unsprayed plants, no recovery was obtained after 1–2 weeks. Under WW conditions, stomatal conductance on the adaxial side of the leaf was significantly higher in PBZ treated plants compared to PBZ untreated ones (Supplementary Figure S2c). However, on the abaxial side of the leaf, stomatal conductance was extremely low but insignificant under both moisture regimes irrespective of PBZ application. As expected, finger millet plants exposed to moisture deficiency lost almost all of their water in their shoots while those sprayed with PBZ maintained up to 80% of their moisture content (Supplementary Figure S2d).

### PBZ Improved the Productivity and Key Agronomic Traits under Drought

The impact of PBZ application on key agronomic traits including grain yield were investigated. Visual observation indicated that PBZ treated plants had deep green leaves compared to PBZ untreated ones. This increased intensity of the green color in PBZ treated plants was also correlated to the significantly higher chlorophyll and carotenoid content in the leaves of PBZ treated plants under both WW and WD conditions (**Table 1**).

**Table 1:** Paclobutrazol improved the productivity and associated traits in tef under moisture scarcity.

	Well-watered (WW)		Water deficit (WD)	
	
	0	100 μM PBZ	0	100 μM PBZ
Chlorophyll *a* (μg/mg DW)	8.30 b	12.27 a	6.03 c	9.25 b
Chlorophyll *b* (μg/mg DW)	3.06 b	4.46 a	1.88 c	3.19 b
Chlorophyll total (μg/mg DW)	11.44 bc	16.73 a	7.45 c	12.44 b
Carotenoids (μg/mg DW)	1.70 b	2.26 a	1.11 c	1.87 b
Days to heading	64.6 a	69.0 b	64.0 a	69.0 b
Days to maturity	89.0 a	91.0 b	93.0 b	95.0 b
Days to senescence	100.2 b	116.0 a	100.8 b	110.8 a
Number of tillers	2.55 b	2.31 a	2.22 c	2.70 b
Numbers of panicles	1.75 b	1.17 b	1.25 b	3.25 a
Plant height (cm)	102.81 a	64.17 c	85.12 b	61.05 c
Shoot biomass (mg/plant)	1278.43 ab	1269.25 ab	807.44 b	1662.40 a
Grain yield (mg/plant)	119.90 b	176.90 a	77.83 b	205.35 a

*Effect of PBZ and moisture stress on photosynthetic pigments (chlorophyll *a*, chlorophyll *b*, carotenoids) and agronomic traits (days to heading, days to maturity, number of tillers, numbers of panicles per plant, plant height, shoot biomass, and grain yield). Means followed by the same letter were not significantly different from each other (*p* = 0.05; *n* = 10)*.

The increased numbers of tillers and numbers of panicles and the reduced plant height due to PBZ application are considered to be positive and desirable effects on plant architecture. The reduction in plant height is preferred in cereal crops particularly in tef as it contributes toward increasing the tolerance of the plant to lodging, the major yielding limiting factor.

Paclobutrazol had a significant influence on days to heading (or flowering), maturity (physiological maturity), and senescence (or harvest maturity) of tef plants. Although the flowering time of PBZ treated plants was longer by 5 days compared to the untreated ones, a wider gap was observed in days to senescence. Depending on moisture regime, PBZ treated plants were delayed by 10–15 days to reach maturity and senescence. As shown in **Table 1**, a significantly higher grain yield (*p* < 0.05) was obtained from PBZ treated plants exposed to both the normal and low soil moisture than from PBZ untreated plants. This high productivity due to PBZ application was related to an increased level of pigments, numbers of tillers and panicles, and the prolonged period to reach maturity and senescence. Interestingly, the effects of PBZ were even more remarkable under limited soil moisture in which over 160% more grain yield was obtained for PBZ treated plants than for their untreated counterparts.

### GA-Deficit Rice Mutants Were Semi-Dwarf and Drought Tolerant

**FIGURE 5:**
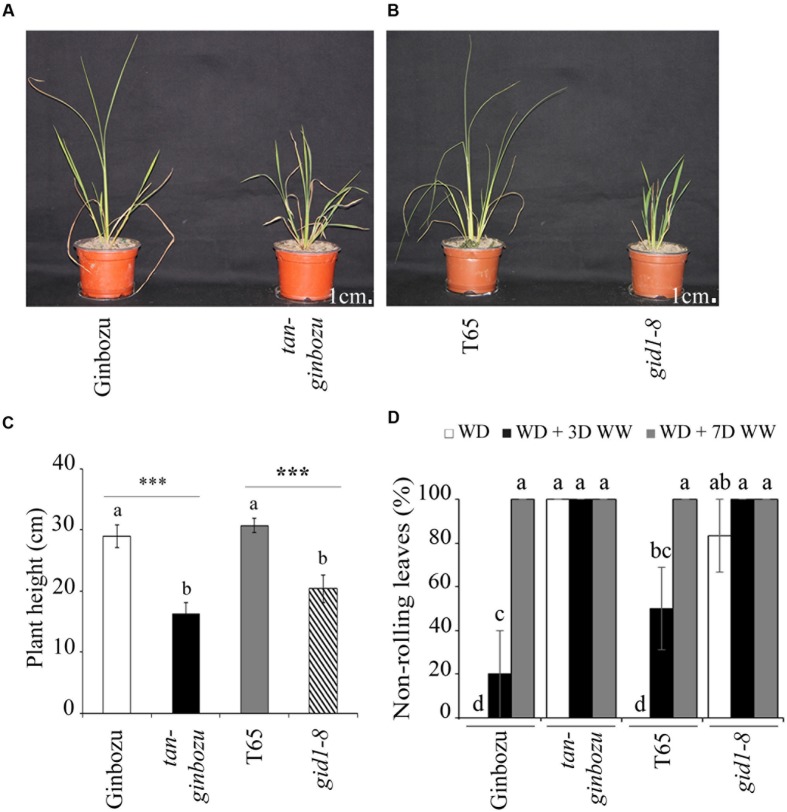
**GA-deficient rice mutants were semi-dwarf and tolerant to moisture deficit**. Visual phenotypes **(A,B)** and plant height **(C)** of semi-dwarf rice mutants and their respective wild types after exposure to moisture deficit. **(D)** Proportion of non-rolling leaf phenotypes under drought (WD), drought followed by 3 days (WD + 3WW) and 7 days (WD + 7WW) of watering. Different letters refer to significant differences among the group of treatments while an asterisk refers to the significant difference between PBZ treated and untreated samples. ^∗∗∗^*p* < 0.001.

Two semi-dwarf rice mutants from the GA biosynthesis pathway, namely *Tan-Ginbozu* and *gid1-8*, exhibited a maximum of 20% leaf rolling while their respective wild types showed up to 100% leaf rolling when exposed to moisture deficit (**Figures 5A–D**). Based on the proportion of non-rolling leaves, an indicator of the recovery of plants from drought stress, the two rice mutants took only 3 days to fully recover while their respective wildtypes required at least 7 days (**Figure 5D**). Under moisture deficit, the stomatal conductance in the abaxial side of *Tan-Ginbozu* and *gid1-8* was significantly lower than their respective wild types (Supplementary Figure S3a). However, the differences in water content were negligible between the wild type and the mutant under both moisture regimes (Supplementary Figure S3b).

### PBZ Did Not Affect the Level of KO2 Transcripts in Tef

**FIGURE 6:**
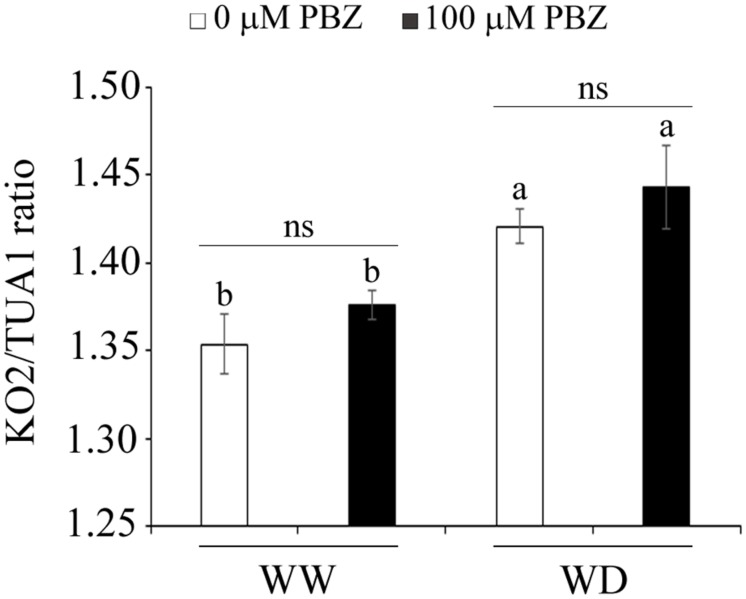
**PBZ did not alter the transcript level of *KO2***. Effect of PBZ and WD on the transcript level of *KO2*, quantified by qRT-PCR. Means followed by the same letter were not significantly different from each other at *p* = 0.05 (*n* = 3).

In order to measure the effect of PBZ on the level of transcripts of the *ent*-kaurene oxidase 2 (*KO2*) gene, the target site of the inhibitor, a quantitative qRT-PCR was done. Although no significant difference in KO2 expression was observed between plants treated with PBZ and control plants, moisture deficiency significantly (*p* < 0.05) increased the expression of this particular gene (**Figure 6**).

### PBZ Protected against Abiotic Stress

**FIGURE 7:**
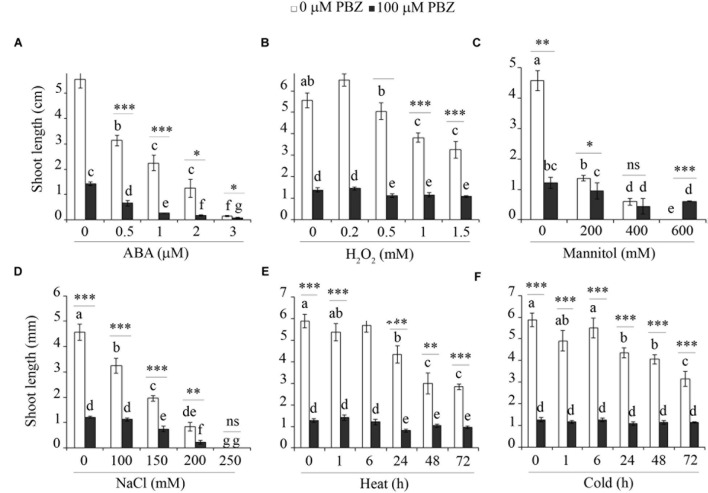
**PBZ treated plants were not influenced by major abiotic stresses**. The shoot length of PBZ treated and PBZ untreated tef plants cv. *Tsedey* exposed to different levels of ABA **(A)**, H_2_O_2_ **(B)**, mannitol **(C)**, NaCl **(D)**, heat **(E)**, and cold **(F)**. Means with different letters refer to the significant difference among the group of treatments while asterisk refers to the significant difference between PBZ treated and PBZ untreated samples (^∗∗∗^*p* < 0.001, ^∗∗^*p* < 0.01, ^∗^*p* < 0.05; ns, no significant difference).

In order to investigate the cross-talk between PBZ and other abiotic stresses, tef seedlings germinated on *in vitro* medium with PBZ were exposed to hormone (e.g., ABA), oxidative stress (H_2_O_2_), salt (NaCl), sugar (mannitol), and extreme temperature (heat and cold). The shoot growth of PBZ untreated tef plants was negatively affected by the increasing level of the six stresses tested (**Figure 7** and Supplementary Figure S4). Although PBZ substantially shortened the shoot of tef plants, further effects due to other abiotic stresses was minimal except for ABA and NaCl. This indicates that plants treated with PBZ were protected against other abiotic stresses. Although PBZ had drastic effects on the growth of tef shoots at the early growth stage, the roots were largely unaffected for both tef genotypes tested (Supplementary Figures S5 and S6). Surprisingly, the responses of PBZ treated and untreated roots to other abiotic stresses were similar for both tef genotypes.

In addition to shoot and root growth, effects on germination, survival and biomass of seedlings were also investigated. Among the six abiotic stresses tested, increasing levels of heat, cold and H_2_O_2_ treatments did not significantly influence the three parameters in both tef genotypes (Supplementary Tables S1 and S2). However, the increasing level of mannitol and NaCl drastically reduced both the germination and the growth of tef seedlings in both tef genotypes irrespective of prior exposure to PBZ.

## Discussion

### Effect of GA Inhibitors on Plant Height and Lodging Tolerance

Synthetic PGRs have a significant effect on plant growth and productivity in terms of total biomass and/or grain yield. The four GA inhibitors, namely CC, DM, MC, and PBZ, used in our experiments contributed to substantial height reduction in tef plants (**Figure 1**). Height reduction due to PBZ was correlated to reduced numbers of internodes and shorter cells compared to untreated plants (**Figure 2**). Earlier studies on several cereals and horticultural crops showed that GA inhibitors, particularly PBZ, were effective in altering the stature of several plant species (Davis et al., 1988; Berova and Zlatev, 2000; Rademacher, 2000; Currey and Lopez, 2010; Kumar et al., 2012).

Paclobutrazol treated tef plants were not only shorter than the untreated plants but also were more tolerant to lodging, the major yield constraint in tef production (**Figure 3**). The reduction in lodging during the two critical growth periods, i.e., the heading and maturity, might be due to the substantial decrease in the height of the plant (**Figure 2A**) and increase in the culm width (**Figure 2B**). These significant changes to the plant architecture reinforce the tef plants against lodging. The positive effect of PBZ on lodging tolerance has been reported earlier for rice (French et al., 1990).

### Effect of Drought and Other Abiotic Stresses on Plant Physiology and Agronomy

The tolerance of PBZ treated plants to moisture scarcity was evaluated using key morphological, physiological, and yield-related parameters. The major morphological indicator for the drought response in cereal crops is the leaf rolling phenotype as has been reported for tef and related Eragrostis species (Balsamo et al., 2006). The higher the proportion of leaf rolling, the less tolerant the plant is to drought. Our results indicate that the percentage of plants with non-rolled leaves (i.e., plants with elevated tolerance to drought) were significantly higher for PBZ treated tef (**Figures 4A,B**) and finger millet (Supplementary Figures S2a,b) than for their untreated counterparts. Similarly, a significantly higher non-rolling leaf rate was also obtained in the case of a GA-deficient rice mutant (**Figure 5D**).

The stomatal conductance of PBZ untreated plants was severely affected under moisture scarcity, while those of PBZ treated ones were unaltered under the two moisture regimes at both the adaxial and abaxial sides of the leaf of tef. Under moisture deficit conditions, stomatal conductance of PBZ treated plants was higher than control plants. Similarly, earlier studies showed that drought tolerant plants maintained higher stomatal conductance than the susceptible ones (Cruz de Cavalho et al., 1998; Turkan et al., 2005). In tef, the application of PBZ delayed the time for the plant to exhibit symptoms associated with moisture deficiency while it facilitated conditions to maintain some moisture for the normal functioning of the plant as postulated in the case of tomato overexpressing AtGAMT1 which catalyze the methylation of active GA (Nir et al., 2014).

Irrespective of the moisture regime, PBZ treated tef plants had greener leaves and significantly higher amounts of chlorophyll and carotenoids than untreated ones (**Table 1**). An increase in photosynthetic pigments was associated with a higher photosynthetic rate and therefore had a positive impact on crop productivity. An earlier study showed that phytyl, a part of the chlorophyll molecule, was synthesized via the same terpenoid pathway as gibberellins (Chaney, 2003). Hence, the higher amount of chlorophyll in PBZ treated plants might be due to the blocking of the gibberellin pathway, which facilitates conditions for the increased chlorophyll production. Increased amounts of these essential pigments due to PBZ application could also be contributing to the significantly higher grain yield and shoot biomass, the two uses of tef for human food and livestock feed, respectively. The tef harvest under limited moisture was remarkable as an additional 50% shoot biomass and 164% grain yield were obtained from PBZ treated plants compared to PBZ untreated ones.

Our findings were in contrast to an earlier study on tef in which the application of PBZ had significant and negative effect on seed yield (Gebre et al., 2012). This extremely low productivity in PBZ treated plants was mainly due to a 98% reduction in plant height (Gebre et al., 2012) compared to only 50% reduction in our study. However, a study in rice indicated that PBZ increased grain yield and quality in rice due to a higher number of spikelets per panicle and a higher grain filling yield (Pan et al., 2013). This exceptionally high productivity under moisture scarcity makes PBZ a chemical with high prospects.

### Links between Abiotic Stress Tolerance and Plant Height

In addition to regulating plant growth, triazoles (including PBZ) are known to protect against abiotic stresses (Fletcher et al., 2000). DELLA protein activity was shown to contribute to osmotic stress tolerance in GA deficient *Arabidopsis* mutants (Achard et al., 2006). DELLA proteins are the major regulators of GA responses and function as suppressors. A GA-GID1-DELLA mechanism that allows for flexible growth regulation in response to environmental conditions has been proposed (Harberd et al., 2009) and may provide an explanation for both the growth and the stress responses. Reduced stature has been linked with abiotic stress response, in species such as *Arabidopsis* and tomato (Nir et al., 2014). DDF1, an AP2-like transcription factor, was shown to reduce the amount of GAs and promotes tolerance to salinity stress (Magome et al., 2004).

In order to test the specificity of the abiotic stress response in tef plants with inhibited GA, we investigated the influence of other abiotic stresses including a hormone (ABA), oxidative stress (H_2_O_2_), sugar (mannitol), salt (NaCl), and temperature (heat and cold) on PBZ treated plants (**Figure 7**). Except for ABA, which is known to be related to drought response in several species (Okuno et al., 2014; Shanker et al., 2014) and NaCl, the other stresses did not substantially alter the response of the tef plants treated with PBZ while they did affect the control plants. Thus the protection against abiotic stress seems to be a general phenomenon rather than specific to drought.

In cereals, little work has been published about the link between abiotic stress tolerance and GA deficiency. PBZ has been shown to protect against heat and paraquat stress in wheat (Kraus and Fletcher, 1994; Gilley and Fletcher, 1997), chilling tolerance in maize and wheat (Pinhero et al., 1997; Berova et al., 2002) and drought tolerance in wheat (Gilley and Fletcher, 1997) although the effects on plant stature were not addressed in these studies. According to Kraus and Fletcher (1994), genes involved in the response of wheat to reactive oxygen species (ROS) were increased in plants treated with PBZ and might be partly responsible for the increased tolerance to abiotic stresses.

The application of PBZ to trees has been shown to retard their growth and increase their tolerance to moisture deficit. According to Chaney (2005), PBZ blocks all three steps that convert *ent*-kaurene to *ent*-kaurenoic acid in the terpenoid pathway which is responsible for the biosynthesis of gibberellins, abscisic acid, phytol and steroids. Hence, an alternative route is taken which produces excessive abscisic acid and in turn enhances the tolerance of the plant to drought.

### Links between Abiotic Stress Tolerance and Plant Architecture in Cereals

Surprisingly, no in-depth studies have shown a connection between lodging and drought tolerance in cereals. A study in barley using GA inhibitors concluded that the shorter the plant, the more tolerant it is to the environmental stresses of heat and paraquat (Sarkar et al., 2004). Another study reported a ‘super dwarf’ wheat mutant that is GA insensitive and has increased drought tolerance (Zhang et al., 2005). Although shorter and thicker internodes in this wheat mutant were mentioned, the observations presented were very preliminary. Interestingly, the authors reported that the mutant showed altered gravitropism and suggested that the mutation might involve auxin for two reasons: firstly, auxin is known to be involved in gravitropism (Moore, 2002), and secondly, the GA and auxin signaling pathways can both regulate growth through common targets (DELLA proteins; Fu and Harberd, 2003). In addition, height reduction and compact lower stalk internodes (as observed here) resulted from the loss of a P-glycoprotein involved in polar auxin transport in maize (Multani et al., 2003).

Investigations are ongoing to determine the mechanism affecting both plant stature and stress tolerance in tef. A transcription factor may be a likely candidate because it would account for the broad effects on stress tolerance and shortened height. In addition, DELLA proteins, antioxidants and members of the GA and auxin pathways are being investigated.

As it is known that PBZ acts on the GA biosynthesis pathway, the expression level of KO2, a key enzyme in early GA biosynthesis, was measured in both control and PBZ treated plants using qPCR. The transcript level of KO2 was not influenced by the application of PBZ (**Figure 6**). This might be due to the fact that PBZ is a competitive inhibitor for KO2 by blocking specifically the three steps in the oxidation of the GA precursor ent-kaurene to ent-kaurenoic acid (Hedden and Graebe, 1985). In other words, the amount of KO2 is unaltered, however, its substrate is blocked by PBZ.

In order to enhance the productivity of crops, cereal breeders are expected to develop crops with multiple desirable traits, especially lodging and drought tolerance as they are the major yield-limiting factors. Although the inhibition of GA synthesis through the application of PBZ resulted in tef and finger millet plants with increased levels of tolerance to both lodging and drought, large scale use of PBZ in farmers’ fields is neither sustainable nor economical. Hence, future research in tef and finger millet needs to focus on developing cultivars deficient in GA biosynthesis similar to those developed for rice (Okuno et al., 2014). This could be achieved through screening natural or mutagenized populations. Phenotypic screening and/or high-throughput screening such as TILLING (Targeting Induced Local Lesions In Genomes) could play a key role in delivering mutant lines with desirable phenotypes. The implementation of TILLING in tef has already produced promising semi-dwarf lines (Tadele et al., 2010; Esfeld et al., 2013) currently undergoing field testing in Ethiopia.

## Conclusion

It is already known that the application of PBZ results in dwarf plants as has been shown in wheat and rice (Crook and Ennos, 1994). However, reduced height does not guarantee reduced lodging and no study has yet indicated that these short mutant plants were also tolerant to drought. Our findings reveal for the first time, the combined beneficial effects of PBZ on the two main yield limiting factors in tef and finger millet, lodging and drought, and have prospects in crop improvement in general and specifically to cereal crops. This double role in height reduction (and lodging tolerance) and drought tolerance was also confirmed in two distinct rice mutants, one in the GA biosynthesis pathway and another in GA signaling. In the future, model and crop plants that are mutated in the GA biosynthesis pathway could be used in breeding programs because of their combined beneficial effects on plant architecture and lodging tolerance as well as abiotic stress tolerance. The time and resources required to introgress each trait individually can be saved by selecting a cultivar with several desirable properties and then crossing to locally adapted and/or high yielding cultivars.

## Author Contributions

SP-W and ZT conceived and designed experiments; SP-W, RB, AR performed experiments; SP-W, GC, and ZT wrote the manuscript; all authors read and approved the final manuscript.

## Conflict of Interest Statement

The authors declare that the research was conducted in the absence of any commercial or financial relationships that could be construed as a potential conflict of interest.

## References

[B1] Achard P. Cheng H. De Grauwe L. Decat J. Schoutteten H. Moritz T. (2006). Integration of plant responses to environmentally activated phytohormonal signals. *Science* 311 91–94. 10.1126/science.1118642 16400150

[B2] Assefa K. Yu J. K. Zeid M. Belay G. Tefera H. Sorrells M. E. (2011). Breeding tef [*Eragrostis tef* (Zucc.) trotter]: conventional and molecular approaches. *Plant Breeding* 130 1–9. 10.1111/j.1439-0523.2010.01782.x

[B3] Balsamo R. A. Willigen C. V. Bauer A. M. Farrant J. (2006). Drought tolerance of selected *Eragrostis* species correlates with leaf tensile properties. *Ann. Bot.* 97 985–991. 10.1093/aob/mcl068 16621860 PMC2803393

[B4] Berova M. Zlatev Z. (2000). Physiological response and yield of paclobutrazol treated tomato plants (*Lycopersicon esculentum* Mill.). *Plant Growth Regul.* 30 117–123. 10.1023/A:1006300326975

[B5] Berova M. Zlatev Z. Stoeva N. (2002). Effect of paclobutrazol on wheat seedlings under low temperature stress. *Bulg. J. Plant Physiol.* 28 75–84.

[B6] Berry P. M. Sterling M. Spink J. H. Baker C. J. Sylvester-Bradley R. Mooney S. J. (2004). Understanding and reducing lodging in cereals. *Adv. Agron.* 84 217–271. 10.1016/S0065-2113(04)84005-7

[B7] Cannarozzi G. Plaza-Wuthrich S. Esfeld K. Larti S. Wilson Y. S. Girma D. (2014). Genome and transcriptome sequencing identifies breeding targets in the orphan crop tef (*Eragrostis tef*). *BMC Genom.* 15:581 10.1186/1471-2164-15-581 PMC4119204 25007843

[B8] Central Statistics Agency [CSA] (2014). *Agricultural Sample Survey for 2013/14 in: Statistical Bulletin 532.* Addis Ababa: Federal Democratic Republic of Ethiopia, Central Statistical Agency.

[B9] Chandrashekar A. (2010). Finger millet *Eleusine coracana*. *Adv. Food Nutr. Res.* 59 215–262. 10.1016/S1043-4526(10)59006-5 20610177

[B10] Chaney W. (2003). Tree growth retardants: arborists discovering new uses for an old tool. *Tree Care Ind. Mag.* 54 2–6.

[B11] Chaney W. R. (2005). *Growth Retardants: A Promising Tool for Managing Urban Trees.* Purdue, IN: FNR-252-W, Purdue Extension, Purdue University.

[B12] Clark R. V. Fedak G. (1977). Effects of chlormequat on plant height, disease development and chemical constituents of cultivars of barley, oats, and wheat. *Can. J. Plant Sci.* 62:6.

[B13] Crook M. J. Ennos A. R. (1994). Stem and root characteristics associated with lodging resistance in 4 winter-wheat cultivars. *J. Agric. Sci.* 123 167–174. 10.1017/S0021859600068428

[B14] Cruz de Cavalho M. H. Laffray D. Louguet P. (1998). Comparison of the physiological responses of *Phaseolus vulgaris* and *Vigna unguiculata* cultivars when submitted to drought conditions. *Environ. Exp. Bot.* 40 197–207. 10.1016/S0098-8472(98)00037-9

[B15] Currey C. J. Lopez R. G. (2010). Paclobutrazol pre-plant bulb dips effectively control height of ’Nellie White’ Easter Lily. *Horttechnology* 20 357–360.

[B16] Davis T. D. Steffens G. L. Sankhla N. (1988). Triazol plant growth regulators. *Hortic. Rev.* 10 151–188.

[B17] Degu E. Adugna A. Tadesse T. Tesso T. (2009). “Genetic resources, breeding and production of millets in Ethiopia,” in *New Approaches to Plant Breeding of Orphan Crops in Africa*, ed. Tadele Z. (Switzerland: University of Bern).

[B18] Esfeld K. Plaza-Wüthrich S. Tadele Z. (2013). “TILLING as a high-throughput technique of tef improvement,” in *Achievements and Prospects of Tef Improvement*, eds Chanyalew A. K. S. Tadele Z. (Bern: EIAR-University of Bern), 53–65.

[B19] Fletcher R.A. Gilley A. Sankhla N. Davis T.D. (2000). “Triazoles as plant growth regulators and stress protectants,” in *Horticultural Reviews*, ed. Janick J. (New York, NY: John Wiley & Sons, Inc.), 55–138.

[B20] French P. Matsuyuki H. Ueno H. (1990). “Paclobutrazol: Control of lodging in Japanese paddy rice,” in *Proceedings of the Conference Held by the Society of Chemical Industry: Pest Management in Rice*, eds Grayson B. T. Green M. B. Copping L. G. (London: Elsevier Applied Science Publishers Ltd.), 474–485.

[B21] Fu X. D. Harberd N. P. (2003). Auxin promotes *Arabidopsis* root growth by modulating gibberellin response. *Nature* 421 740–743. 10.1038/nature01387 12610625

[B22] Gebre E. Schlüter U. Hedden P. Kunert K. (2012). Gibberellin biosynthesis inhibitors help control plant height for improving lodging resistance in *E. tef (Eragrostis tef)*. *J. Crop Improv.* 26 375–388. 10.1080/15427528.2011.646056

[B23] Gilley A. Fletcher R. A. (1997). Relative efficacy of paclobutrazol, propiconazole and tetraconazole as stress protectants in wheat seedlings. *Plant Growth Regul.* 21 169–175. 10.1023/A:1005804717016

[B24] Harberd N. P. Belfield E. Yasumura Y. (2009). The Angiosperm gibberellin-GID1-DELLA growth regulatory mechanism: how an “inhibitor of an inhibitor” enables flexible response to fluctuating environments. *Plant Cell* 21 1328–1339. 10.1105/tpc.109.066969 19470587 PMC2700538

[B25] Hedden P. (2003). The genes of the green revolution. *Trends Genet.* 19 5–9. 10.1016/S0168-9525(02)00009-4 12493241

[B26] Hedden P. Graebe J. E. (1985). Inhibition of gibberellin biosynthesis by paclobutrazol in cell-free homogenates of *Cucurbita-Maxima* Endosperm and *Malus pumila* Embryos. *J. Plant Growth Regul.* 4 111–122. 10.1007/BF02266949

[B27] Kraus T. E. Fletcher R. A. (1994). Paclobutrazol protects wheat seedlings from heat and paraquat injury: is detoxification of active oxygen involved? *Plant Cell Physiol.* 35 45–52.

[B28] Kumar S. Ghatty S. Satyanarayana J. Guha A. Chaitanya B. Reddy A. R. (2012). Paclobutrazol treatment as a potential strategy for higher seed and oil yield in field-grown *Camelina sativa* L. Crantz. *BMC Res. Notes* 5:137 10.1186/1756-0500-5-137 PMC3320555 22410213

[B29] Lichtenthaler H. K. (1987). Chlorophyll fluorescence signatures of leaves during the autumnal chlorophyll breakdown. *J. Plant Physiol.* 131 101–110. 10.1016/S0176-1617(87)80271-7

[B30] Livak K. J. Schmittgen T. D. (2001). Analysis of relative gene expression data using real-time quantitative PCR and the 2(T)(-Delta Delta C) method. *Methods* 25 402–408. 10.1006/meth.2001.1262 11846609

[B31] Lopes M. S. Araus J. L. Van Heerden P. D. R. Foyer C. H. (2011). Enhancing drought tolerance in C-4 crops. *J. Exp. Bot.* 62 3135–3153. 10.1093/jxb/err105 21511912

[B32] Magome H. Yamaguchi S. Hanada A. Kamiya Y. Oda K. (2004). dwarf and delayed-flowering 1 a novel *Arabidopsis* mutant deficient in gibberellin biosynthesis because of overexpression of a putative AP2 transcription factor. *Plant J.* 37 720–729. 10.1111/j.1365-313X.2003.01998.x 14871311

[B33] Mccarty J. C. Hedin P. A. (1994). Effects of 11-Dimethylpiperidinium Chloride on the yields, agronomic traits, and allelochemicals of cotton (*Gossypium-hirsutum* L), a 9-Year Study. *J. Agric. Food Chem.* 42 2302–2304.

[B34] Mengistu D. K. (2009). The influence of soil water deficit imposed during various developmental phases on physiological processes of tef (*Eragrostis tef*). *Agric. Ecosyst. Environ.* 132 283–289. 10.1016/j.agee.2009.04.013

[B35] Moore I. (2002). Gravitropism: lateral thinking in auxin transport. *Curr. Biol.* 12 R452–R454. 10.1016/S0960-9822(02)00943-0 12121635

[B36] Multani D. S. Briggs S. P. Chamberlin M. A. Blakeslee J. J. Murphy A. S. Johal G. S. (2003). Loss of an MDR transporter in compact stalks of maize br2 and sorghum dw3 mutants. *Science* 302 81–84. 10.1126/science.1086072 14526073

[B37] Murashige T. Skoog F. (1962). A revised medium for rapid growth and bio assays with tobacco tissue cultures. *Physiol. Plant.* 15 473–497. 10.1111/j.1399-3054.1962.tb08052.x

[B38] NAP (1996). *Lost Crops of Africa, Volume I: Grains.* Washington, DC: National Academy Press.

[B39] Nir I. Moshelion M. Weiss D. (2014). The *Arabidopsis* GIBBERELLIN METHYL TRANSFERASE 1 suppresses gibberellin activity, reduces whole-plant transpiration and promotes drought tolerance in transgenic tomato. *Plant Cell Environ.* 37 113–123. 10.1111/pce.12135 23668385

[B40] Okuno A. Hirano K. Asano K. Takase W. Masuda R. Morinaka Y. (2014). New approach to increasing rice lodging resistance and biomass yield through the use of high gibberellin producing varieties. *PLoS ONE* 9:e86870 10.1371/journal.pone.0086870 PMC3929325 24586255

[B41] Otoole J. C. Cruz R. T. (1980). Response of leaf water potential, stomatal-resistance, and leaf rolling to water-stress. *Plant Physiol.* 65 428–432. 16661206 10.1104/pp.65.3.428 PMC440347

[B42] Pan S. Rasul F. Li W. Tian H. Mo Z. Duan M. (2013). Roles of plant growth regulators on yield, grain qualities and antioxidant enzyme activities in super hybrid rice (*Oryza sativa* L.). *Rice* 6:9 10.1186/1939-8433-6-9 PMC4883720 24280625

[B43] Pinhero R. G. Rao M. V. Paliyath G. Murr D. P. Fletcher R. A. (1997). Changes in activities of antioxidant enzymes and their relationship to genetic and Paclobutrazol-Induced chilling tolerance of maize seedlings. *Plant Physiol.* 114 695–704. 12223737 10.1104/pp.114.2.695 PMC158354

[B44] Provost C. Jobson E. (2014). Move over quinoa, Ethiopia’s teff poised to be next big super grain. *Guardian.*

[B45] Rademacher W. (2000). Growth retardants: effects on gibberellin biosynthesis and other metabolic pathways. *Annu. Rev. Plant Physiol. Plant Mol. Biol.* 51 501–531. 10.1146/annurev.arplant.51.1.501 15012200

[B46] Sanvicente P. Lazarevitch S. Blouet A. Guckert A. (1999). Morphological and anatomical modifications in winter barley culm after late plant growth regulator treatment. *Eur. J. Agron.* 11 45–51. 10.1016/S1161-0301(99)00017-9

[B47] Sarkar S. Perras M. R. Falk D. E. Zhang R. C. Pharis R. P. Fletcher R. A. (2004). Relationship between gibberellins, height, and stress tolerance in barley (*Hordeum vulgare* L.) seedlings. *Plant Growth Regul.* 42 125–135. 10.1023/B:GROW.0000017492.56792.64

[B48] Shanker A. K. Maheswari M. Yadav S. K. Desai S. Bhanu D. Attal N. B. (2014). Drought stress responses in crops. *Funct. Integr. Genom.* 14 11–22. 10.1007/s10142-013-0356-x 24408129

[B49] Spaenij-Dekking L. Kooy-Winkelaar Y. Koning F. (2005). The ethiopian cereal tef in celiac disease. *N. Engl. J. Med.* 353 1748–1749. 10.1056/NEJMc051492 16236752

[B50] Tadele Z. (2016). “Drought adaptation in millets,” in *Abiotic and Biotic Stress in Plants: Recent Advances and Future Perspectives*, eds Shanker A. Shanker C. (Rijeka: InTech), 639–662.

[B51] Tadele Z. Assefa K. (2012). Increasing food production in Africa by boosting the productivity of understudied crops. *Agronomy* 2 240–283. 10.3390/agronomy2040240

[B52] Tadele Z. Mba C. Till B.J. (2010). “TILLING for mutations in model plants and crops,” in *Molecular Techniques in Crop Improvement*: 2nd Edn. eds Jain S. M. Brar D. S. (Berlin: Springer), 307–332.

[B53] Turkan I. Bor M. Ozdemir F. Koca H. (2005). Differential responses of lipid peroxidation and antioxidants in the leaves of drought-tolerant *P. acutifolius* Gray and drought-sensitive *P. vulgaris* L. subjected to polyethylene glycol mediated water stress. *Plant Sci.* 168 223–231. 10.1016/j.plantsci.2004.07.032

[B54] van Delden S. H. Vos J. Ennos A. R. Stomph T. J. (2010). Analysing lodging of the panicle bearing cereal teff (*Eragrostis tef*). *New Phytol.* 186 696–707. 10.1111/j.1469-8137.2010.03224.x 20345637

[B55] WSR (2015). *ImageJ.* Available at: http://rsbweb.nih.gov/ij/index.html (accessed Febreuary 4 2016).

[B56] Yami A. (2013). “Tef straw: a valuable feed resource to improve animal production and productivity,” in *Achievements and Prospects of Tef Improvement*, eds Assefa K. Chanyalew S. Tadele Z. (Bern: EIAR-Uni. Bern), 233–251.

[B57] Zhang X. Chen X. Wu Z. Zhang X. Huang C. Cao M. (2005). A dwarf wheat mutant is associated with increased drought resistance and altered responses to gravity. *Afr. J. Biotechnol.* 4 1054–1057.

