# Supplementary material for: Gibberellin Deficiency Confers Both Lodging and Drought Tolerance in Small Cereals
Source: Front Plant Sci. 2016 May 13;7:643. doi: 10.3389/fpls.2016.00643 (PMC4865506; doi:10.3389/fpls.2016.00643)
Supplement: Supplementary file 1 [file Presentation_1.PDF]

Supplemental Data

Gibberellin Deficiency Confers Both Lodging and Drought Tolerance in Small Cereals

Sonia Plaza-Wüthrich[1], Regula Blösch[1], Abiel Rindisbacher[1], Gina cannarozzi[1], Zerihun Tadele[1, 2, *]

[1]Institute of Plant Sciences, University of Bern, Altenbergrain 21, CH-3013 Bern, Switzerland; [2]Addis Ababa University, Institute of Biotechnology, P.O. Box 32853, Addis Ababa, Ethiopia; [*]Corresponding Author

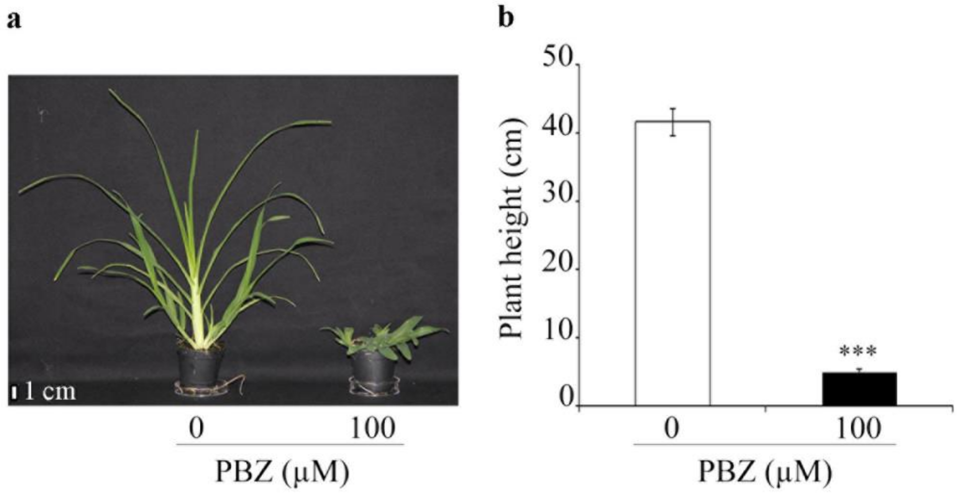

Figure S1. PBZ substantially reduced the height of finger millet plants. The phenotype (a) and height (b) of PBZ treated and PBZ untreated plants.

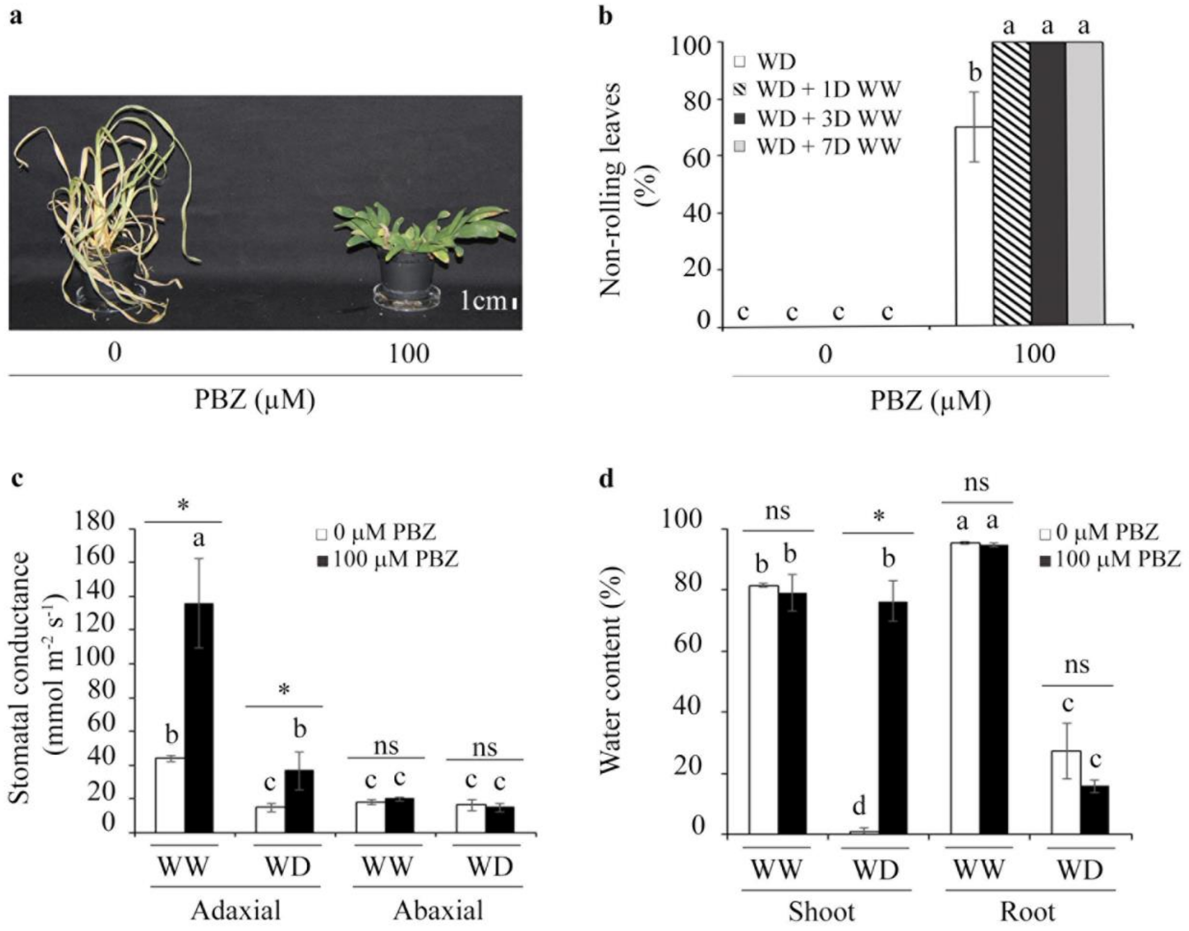

Figure S2. PBZ enhanced the tolerance of finger millet to drought through morphological and physiological changes. (a) response of PBZ treated plants exposed to moisture deficit, (b) proportion of non-rolling leaves after treatment with PBZ and exposure to drought, (c) stomatal conductance of PBZ treated and PBZ untreated finger millet plants under normal watering (WW) and water deficit (WD) conditions. (d) water content of PBZ treated and PBZ untreated plants. Different letters refer to significant differences among the group of treatments while * refers to the significant difference between PBZ treated and untreated samples. *** = $P<0.001$, ** = $P<0.01$, * = $P<0.05$; ns, no significant difference.

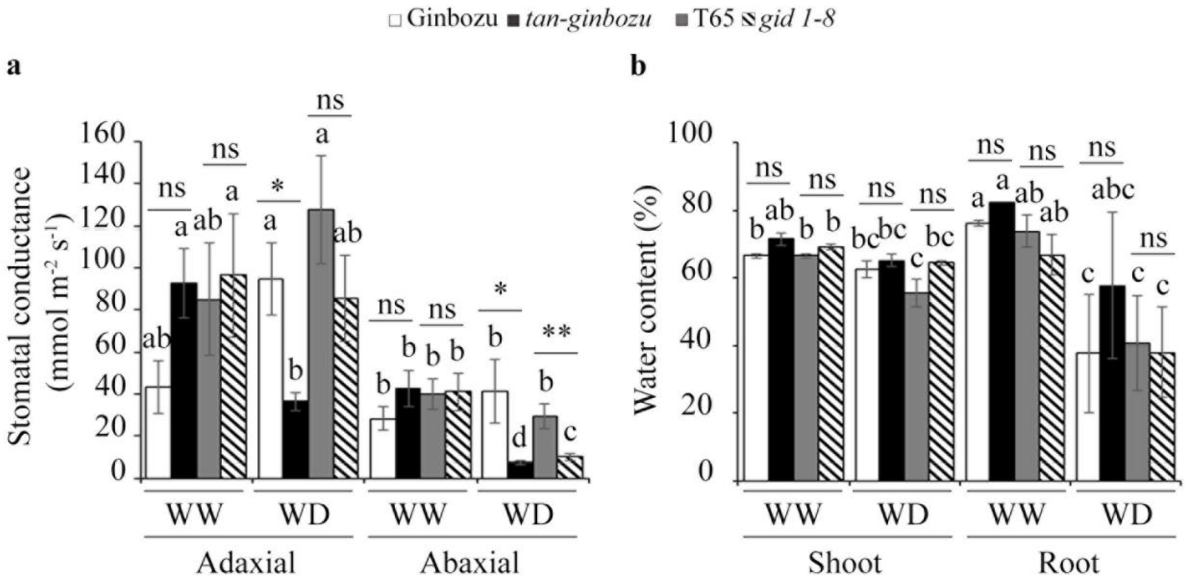

Figure S3. GA-deficient rice mutants increased drought tolerance under moisture deficient conditions. (a) stomatal conductance of the adaxial and abaxial sides of leaves, (b) shoot and root water content of GA-deficient rice lines under normally watered (WW) and drought (WD) conditions. Different letters refer to significant differences among the group of treatments while * refers to the significant difference between PBZ treated and untreated samples. *** = P<0.001, ** = P<0.01, * = P<0.05; ns, no significant difference for normally watered (WW) and drought (WD)

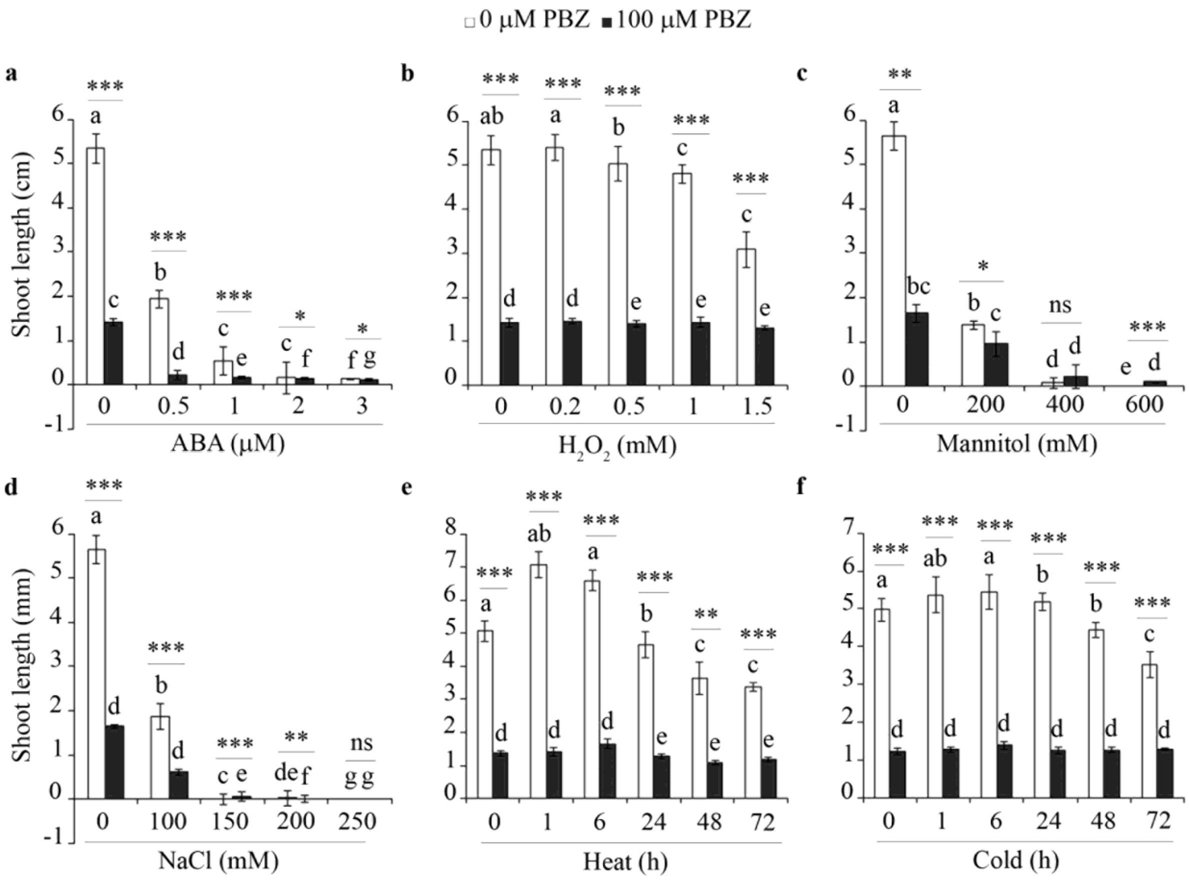

Figure S4. The shoot length of PBZ treated and PBZ untreated tef plants cv *Dukem* exposed to different levels of ABA (a), H_2O_2 (b), mannitol (c), NaCl (d), heat (e), and cold (f). Means with different letters refer to the significant difference among the group of treatments while * refers to a significant difference between PBZ treated and PBZ untreated samples (*** = P<0.001, ** = P<0.01, * = P<0.05; ns, no significant difference).

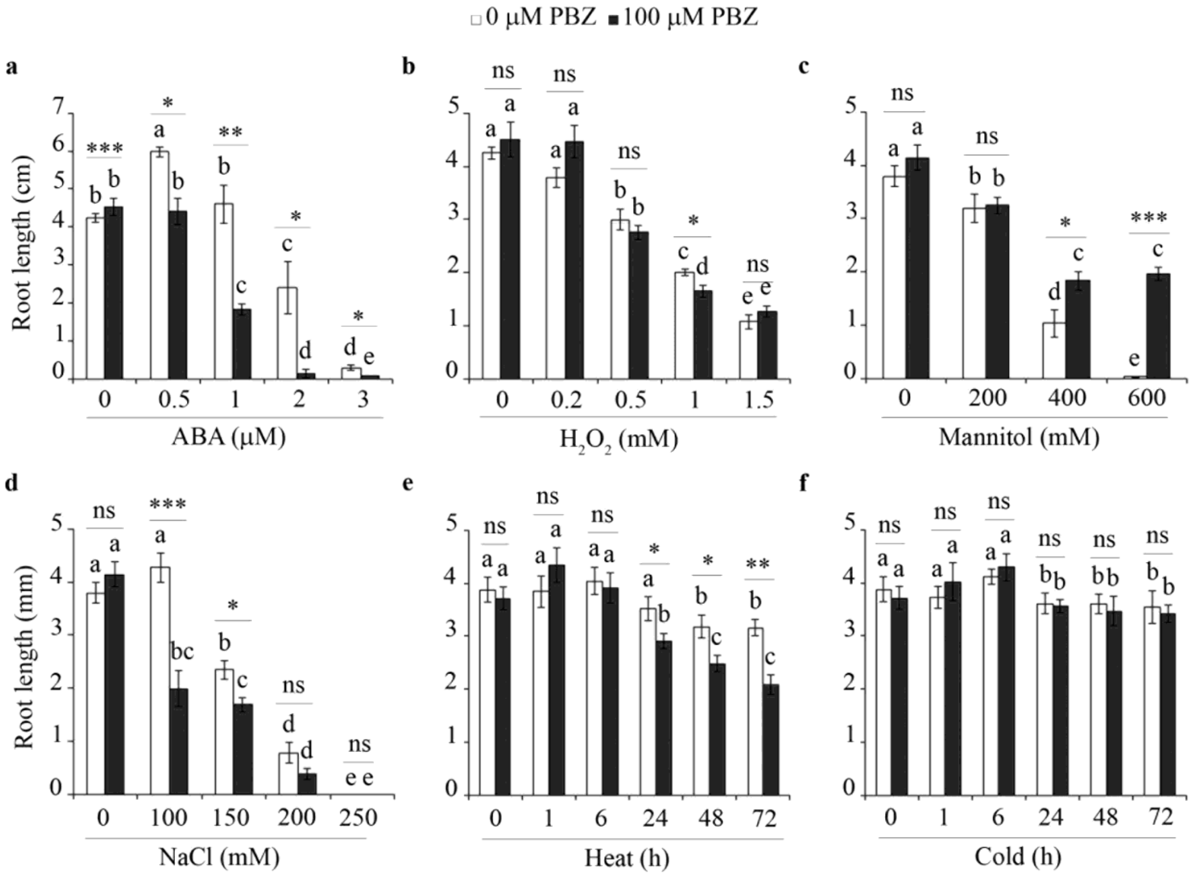

Figure S5. The root length of PBZ treated and PBZ untreated tef plants cv *Tsedey* exposed to different levels of ABA (a), H_2O_2 (b), mannitol (c), NaCl (d), heat (e), and cold (f). Means with different letters refer to a significant difference among the group of treatments while * refers to a significant difference between PBZ treated and PBZ untreated samples (*** = P<0.001, ** = P<0.01, * = P<0.05; ns, no significant difference).

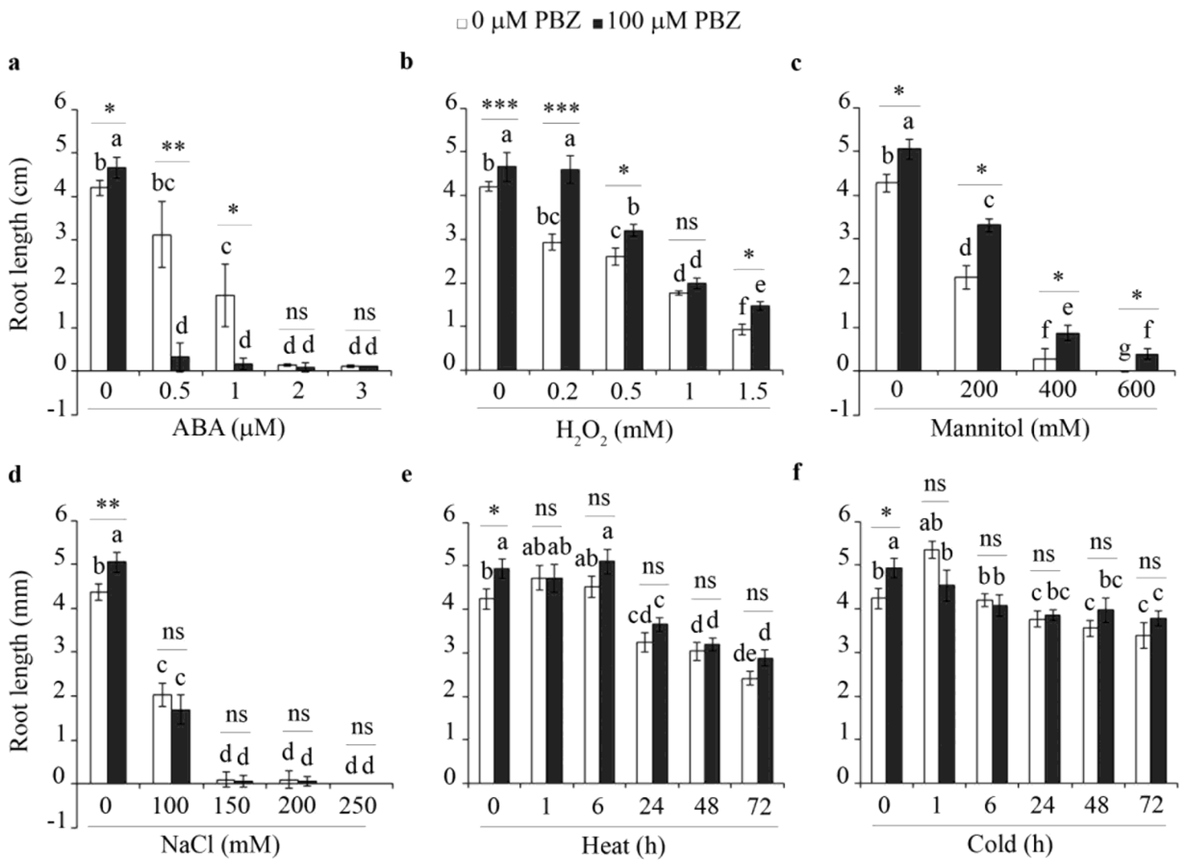

Figure S6. The root length of PBZ treated and PBZ untreated tef plants cv *Dukem* exposed to different levels of ABA (a), H_2O_2 (b), mannitol (c), NaCl (d), heat (e), and cold (f). Means with different letters refer to a significant difference among the group of treatments while * refers to a significant difference between PBZ treated and PBZ untreated samples (*** = P<0.001, ** = P<0.01, * = P<0.05; ns, no significant difference).

Table S1. Effect of different levels of abiotic stresses on germination, survival and shoot biomass of tef cv. *Tsedey* treated by 10 µM PBZ. Means in each column followed by the same letter were not significantly different (P = 0.05) for each type of stress while an asterisk indicates a significant difference between PBZ untreated and PBZ treated samples.

Abiotic stress	Amount or time	PBZ (µM) –>	Germination (%)		Survival (%)		Dry biomass (mg)	
			0	10	0	10	0	10
ABA (µM)	0		100.00 a	100.00 a	100.00 a	100.00 a	0.74 b	0.82 a
	0.5		100.00 a	100.00 a	100.00 a	63.64 *b	1.12 a	0.21 *b
	1		100.00 a	100.00 a	92.31 a	23.08 *c	0.23 c	0.08 *c
	2		91.67 a	100.00 a	54.54 b	8.33 *c	0.21 c	0.08 *c
	3		83.33 b	91.67 *a	0.08 c	0.00 c	0.08 c	0.01 c
H_2O_2 (mM)	0		100.00 a	100.00 a	100.00 a	100.00 a	0.74 b	0.82 a
	0.2		100.00 a	100.00 a	100.00 a	100.00 a	1.13 a	0.51 *b
	0.5		100.00 a	100.00 a	100.00 a	100.00 a	0.98 b	0.52 b
	1		100.00 a	100.00 a	100.00 a	100.00 a	0.55 c	0.42 c
	1.5		100.00 a	100.00 a	100.00 a	100.00 a	0.57 c	0.39 c
Mannitol (mM)	0		100.00 a	100.00 a	100.00 a	100.00 a	1.12 a	1.06 a
	200		92.31 ab	100.00 a	92.31 a	100.00 a	0.69 b	0.33 *b
	400		83.33 b	100.00 *a	66.67 b	84.62 a	0.24 c	0.47 *b
	600		0.00 c	84.62 *b	0.00 c	84.62 *a	0.00 d	0.47 *b
NaCl (mM)	0		100.00 a	100.00 a	100.00 a	100.00 a	1.12 a	1.06 a
	100		91.67 a	92.31 b	91.67 a	92.31 b	0.76 b	0.41 *b
	150		91.67 a	84.62 b	91.67 a	71.43 *b	0.46 c	0.45 *b
	200		23.08 b	33.33 c	23.08 b	33.33 c	0.28 d	0.01 *c
	250		16.67 b	25.00 c	0.00 c	0.00 d	0.00 e	0.00 c
Heat (h)	0		100.00 a	100.00 a	100.00 a	100.00 a	0.87 a	0.69 a
	1		100.00 a	100.00 a	100.00 a	100.00 a	0.57 c	0.67 a
	6		100.00 a	100.00 a	100.00 a	100.00 a	0.95 a	0.83 a
	24		100.00 a	100.00 a	100.00 a	100.00 a	0.81 a	0.75 a
	48		100.00 a	100.00 a	100.00 a	100.00 a	0.75 b	0.47 *b
	72		100.00 a	100.00 a	100.00 a	100.00 a	0.69 bc	0.15 *c
Cold (h)	0		100.00 a	100.00 a	100.00 a	100.00 a	0.87 a	0.69 a
	1		100.00 a	100.00 a	100.00 a	100.00 a	0.81 a	0.64 a
	6		100.00 a	100.00 a	100.00 a	100.00 a	0.85 a	0.67 *a
	24		100.00 a	100.00 a	100.00 a	100.00 a	0.79 ab	0.47 *b
	48		100.00 a	100.00 a	100.00 a	100.00 a	0.47 b	0.37 *b
	72		100.00 a	100.00 a	100.00 a	100.00 a	0.40 b	0.37 b

Table S2. Effect of different levels of abiotic stresses on germination, survival and shoot biomass of tef cv. *Dukem* treated by 10 µM PBZ. Means in each column followed by the same letter were not significantly different (P = 0.05) for each type of stress while an asterisk indicates a significant difference between PBZ untreated and PBZ treated samples.

Abiotic stress	Amount or time	PBZ (µM) –>	Germination (%)		Survival (%)		Dry weight (mg)	
			0	10	0	10	0	10
**ABA (µM)**	**0**		100.00 a	100.00 a	100.00 a	100.00 a	0.88 b	0.92 a
	**0.5**		100.00 a	100.00 a	91.67 a	8.33 *b	1.81 a	0.22 *b
	**1**		91.67 a	100.00 a	27.27 b	0.00 *b	0.12 c	0.22 *b
	**2**		90.00 a	100.00 a	9.09 c	0.00 *b	0.15 c	0.24 *b
	**3**		90.00 a	90.00 a	0.00 c	0.00 b	0.15 c	0.24 *b
**H_2O_2 (mM)**	**0**		100.00 a	100.00 a	100.00 a	100.00 a	0.88 a	0.92 a
	**0.2**		100.00 a	100.00 a	100.00 a	100.00 a	0.71 a	0.63 a
	**0.5**		100.00 a	100.00 a	100.00 a	100.00 a	0.69 a	0.64 a
	**1**		100.00 a	100.00 a	100.00 a	100.00 a	0.70 a	0.48 *b
	**1.5**		100.00 a	100.00 a	100.00 a	100.00 a	0.44 b	0.42 b
**Mannitol (mM)**	**0**		100.00 a	100.00 a	100.00 a	100.00 a	1.27 a	1.37 a
	**200**		100.00 a	91.67 a	91.67 a	91.67 a	0.76 b	0.36 *b
	**400**		75.00 b	75.00 b	8.33 b	16.67 b	0.28 c	0.34 b
	**600**		0.00 c	66.67 *b	0.00 c	16.67 *c	0.00 d	0.24 *b
**NaCl (mM)**	**0**		100.00 a	100.00 a	100.00 a	100.00 a	1.27 a	1.37 a
	**100**		80.00 a	66.67 b	53.85 b	58.33 b	0.50 b	0.41 b
	**150**		25.00 b	46.15 b	10.00 c	8.33 c	0.08 c	0.04 c
	**200**		21.43 b	8.33 *c	8.33 c	0.00 *d	0.00 c	0.00 c
	**250**		0.00 c	8.33 *c	0.00 d	0.00 d	0.00 c	0.00 c
**Heat (h)**	**0**		100.00 a	100.00 a	100.00 a	100.00 a	1.20 a	1.05 a
	**1**		100.00 a	100.00 a	100.00 a	100.00 a	1.18 a	0.63 *b
	**6**		100.00 a	100.00 a	100.00 a	100.00 a	1.18 a	0.99 a
	**24**		100.00 a	100.00 a	100.00 a	100.00 a	0.84 b	1.05 *a
	**48**		100.00 a	100.00 a	100.00 a	100.00 a	0.79 b	0.85 ab
	**72**		100.00 a	100.00 a	100.00 a	100.00 a	0.78 b	0.85 ab
**Cold (h)**	**0**		100.00 a	100.00 a	100.00 a	100.00 a	1.20 a	1.05 a
	**1**		100.00 a	100.00 a	100.00 a	100.00 a	1.06 ab	0.51 *b
	**6**		100.00 a	100.00 a	100.00 a	100.00 a	0.66 b	0.53 b
	**24**		100.00 a	100.00 a	100.00 a	100.00 a	0.52 b	0.46 b
	**48**		100.00 a	100.00 a	100.00 a	100.00 a	0.55 b	0.37 b
	**72**		100.00 a	100.00 a	100.00 a	100.00 a	0.54 b	0.38 b
